# What Are the Reasons for Poor Uptake of HIV Testing among Patients with TB in an Eastern India District?

**DOI:** 10.1371/journal.pone.0055229

**Published:** 2013-03-01

**Authors:** Bipra Bishnu, Sudipto Bhaduri, Ajay M. V. Kumar, Eleanor S. Click, Vineet Kumar Chadha, Srinath Satyanarayana, Sreenivas Achutan Nair, Devesh Gupta, Quazi T. Ahmed, Silajit Sarkar, Durba Paul, Puneet Dewan

**Affiliations:** 1 Office of the World Health Organization (WHO) Representative in India, WHO Country Office, New Delhi, India; 2 District Tuberculosis Centre, Ministry of Health and Family Welfare, Government of West Bengal, Kolkata, India; 3 International Union against Tuberculosis and Lung Diseases (The Union), South East Asia Regional Office, New Delhi, India; 4 Division of Tuberculosis Elimination, Centers for Disease Control and Prevention, Atlanta, Georgia, United States of America; 5 National Tuberculosis Institute, Bangalore, India; 6 Central TB Division, Directorate General of Health Services, Ministry of Health and Family Welfare, Government of India, New Delhi, India; London School of Hygiene and Tropical Medicine, United Kingdom

## Abstract

**Background:**

National policy in India recommends HIV testing of all patients with TB. In West Bengal state, only 28% of patients with TB were tested for HIV between April-June, 2010. We conducted a cross-sectional survey to understand patient, provider and health system related factors associated with low uptake of HIV testing among patients with TB.

**Methods:**

We reviewed TB and HIV program records to assess the HIV testing status of patients registered for anti-TB treatment from July-September 2010 in South-24-Parganas district, West Bengal, assessed availability of HIV testing kits and interviewed a random sample of patients with TB and providers.

**Results:**

Among 1633 patients with TB with unknown HIV status at the time of diagnosis, 435 (26%) were tested for HIV within the intensive phase of TB treatment. Patients diagnosed with and treated for TB at facilities with co-located HIV testing services were more likely to get tested for HIV than at facilities without [RR = 1.27, (95% CI 1.20–3.35)]. Among 169 patients interviewed, 67 reported they were referred for HIV testing, among whom 47 were tested. During interviews, providers attributed the low proportion of patients with TB being referred and tested for HIV to inadequate knowledge among providers about the national policy, belief that patients will not test for HIV even if they are referred, shortage of HIV testing kits, and inadequate supervision by both programs.

**Discussion:**

In West Bengal, poor uptake of HIV testing among patients with TB was associated with absence of HIV testing services at sites providing TB care services and to poor referral practices among providers. Comprehensive strategies to change providers’ beliefs and practices, decentralization of HIV testing to all TB care centers, and improved HIV test kit supply chain management may increase the proportion of patients with TB who are tested for HIV.

## Introduction

Early HIV testing for all patients with Tuberculosis (TB) followed by provision of cotrimoxazole prophylaxis therapy (CPT) and early initiation of antiretroviral therapy (ART) for those found to be HIV positive has been shown to reduce mortality among patients with both TB disease and HIV infection (hereafter referred to as TB/HIV) [1]. India has the highest absolute number of TB cases and the second highest number of TB/HIV cases in the world [2], [3]. The initial 2007 national framework for joint collaboration between the Revised National TB Control Program (RNTCP) and the National AIDS Control Program (NACP) promoted selective referral of patients with “high risk” behavior (e.g. truck drivers or sex-workers who were not responding to anti-TB treatment) for HIV counseling and testing at the nearest HIV testing center (Integrated Counseling and Testing Centre, ICTC). ICTC are managed by the NACP and are either ‘stand-alone’ HIV-testing facilities or located adjacent to clinical care facilities. The framework was revised in 2008 to recommend provider-initiated HIV testing and counseling (PITC) for all patients registered for TB treatment as recommended by WHO and UNAIDS [4]. The policy of referring all patients diagnosed with TB to the nearest HIV testing center and getting them tested for HIV is being scaled-up in a phased manner with a vision of achieving nationwide coverage by the end of 2012 [5]. HIV testing is the entry point to the HIV care continuum for people with TB who have undiagnosed HIV and is provided free of cost by the NACP [6]. Between April and June 2010, in 16 states of India implementing PITC, 61% (121,688/199,944) of patients registered for TB treatment had documented HIV status [3].

In the eastern Indian state of West Bengal, preparatory activities for implementation of PITC for patients with TB were completed by March 2010. These activities included sensitization and training of TB program managers and all levels of medical and para-medical staff using standard training modules developed by both the national TB and HIV programs. However, in the first quarter of implementation of PITC (April through June, 2010), only 28% (7,936/28,367) of patients with TB in West Bengal had documented HIV status, ranging from 0–40% among the 19 districts of the state [3]. We aimed to understand the factors associated with poor uptake of HIV testing among patients with TB in order to facilitate necessary corrective actions. The present study was carried out in South 24 Parganas, one of the largest districts in West Bengal State, in order to assess patient, provider, and health system related factors for poor uptake of HIV testing during the initial intensive phase of patients’ anti-TB treatment.

## Methods

### Ethics Statement

All records reviewed as part of this study are routinely collected and documented by RNTCP and NACP in India. Participation in interviews was entirely voluntary and we obtained written informed consent from all adult participants or from a parent of participants aged less than 15 years. Interviewers were blinded to the HIV testing status of participants. A total of three interview attempts were made for patients who could not be located initially. The entire study protocol was reviewed and approved by Institutional Ethics Committee of National TB Institute, Bangalore, India and Ethics Advisory Group of the International Union against TB and Lung Disease. Approval to conduct the study was obtained from Central TB Division and the State Health department.

### Study Design

This was a cross sectional study and the study procedures encompassed a mix of quantitative and qualitative data collection methods: 1) record review (from both TB and HIV programs) for all patients registered for TB treatment in South Parganas 24 District, 2) review of records documenting TB/HIV training for individual health personnel of the district, 3) review of HIV test-kit stock registers, 4) interview of a subset of patients with TB and 5) interview and focus group discussion (FGD) with TB care providers and HIV care providers.

### Setting

South 24 Parganas District (population 7.7 million) is primarily a rural district of West Bengal with a HIV sero-prevalence of more than 1% among antenatal women [6]. Prevalence of HIV among people with TB is not known. TB control program services are available through a decentralized network of 102 general health care facilities. In addition, patients also frequently utilize health facilities in the city of Kolkata, located in an adjacent district, and subsequently complete treatment at one of the health care facility in South 24 Parganas districts. In accordance with RNTCP guidelines [7], the 102 South 24 Parganas facilities are grouped into 15 sub-district level TB program management units (TU) so each TU has 4 to 12 health care facilities. Patients with TB are registered within one month of treatment initiation, at one of the 15 TUs and are treated with standardized treatment regimens administered under direct observation. TB treatment consists of intensive phase (IP) for 2 to 3 months and continuation phase for 4 to 5 months. Each TU has a TB register maintained by a TB treatment supervisor to document details of patients started on anti-TB treatment. Register data also include date and place of diagnosis, date and place of treatment for TB, HIV status, and outcome of treatment. Each TB treatment supervisor works under the supervision and guidance of a Medical Officer of TB Control (MOTC). Of 102 health institutions that provide TB treatment services, 14 have a co-located HIV testing center called the Integrated Counseling and Testing Centre (ICTC). Each of the remaining 88 health institutions is affiliated with one of these 14 ICTCs in order to provide access to HIV testing services for their patients. Each ICTC is staffed by a counsellor and a lab-technician and is monitored by the NACP. HIV testing consists of one rapid test which if found positive is followed by two confirmatory rapid tests [8]. Details of each client (name, age, sex and address) attending the ICTC are documented in an identification register referred to as the Person Identification Digit (PID) register. The HIV test results are documented in a separate ICTC register using the unique PID number for each patient from the PID register.

### Study Population and Study Period

1. The study population included all patients with TB registered for treatment under RNTCP in South 24 Parganas district from 1st July to 30^th^ September 2010. Patients with “known HIV status” (defined as any prior positive test result or as a negative result for a test performed within six months prior to diagnosis of TB) were excluded from the study.
2. All health care providers from TB and HIV services in the district formed the sampling frame for provider interviews.

The study was conducted during the period July 2010– March 2011.

### Data Source, Sample Size, Sampling Method and Variables

The primary outcome variable was *failure to be tested for HIV* by the end of IP of anti-TB treatment. National guidelines recommend referral for HIV testing as soon as possible after diagnosis of TB; hence, in the present study, the time limit for defining the outcome variables was the end of the IP of treatment (i.e. 2 months for new cases and 3 months for retreatment cases). Multiple methods of data collection were used including the following quantitative and qualitative components:

#### Record review- quantitative component

We reviewed both TB and HIV program records. We used structured data collection sheets to abstract data for all patients registered at the South 24 Parganas TUs. We documented the following variables from the TB register: age, sex, history of previous TB treatment, classification of TB and health facility where TB was diagnosed and got treatment respectively. We reviewed PID and ICTC registers at the ICTCs linked to the health facilities where patients with TB were diagnosed and where patients received treatment, if diagnosis and treatment occurred at different facilities linked to different ICTC. For patients with TB treatment records that indicated they were diagnosed with TB at a facility outside of South 24 Parganas (e.g., in Kolkata) we also reviewed the PID and ICTC registers at the ICTC linked to the facility where the patient was diagnosed. We linked TB treatment register and PID register data using patient name, age and address to determine whether a patient with TB attended the ICTC linked to either the site of TB diagnosis or treatment. If the name was found in the PID register, the ICTC register was searched to determine whether the patient was tested for HIV. A patient with TB was considered to have been tested for HIV if his or her name and test results could be located in PID and ICTC registers. We noted the date of testing for HIV in our structured data collection sheet. If a matching patient record was not found in the ICTC register for dates corresponding to the intensive phase of TB treatment, we searched for matching records from the 6 months prior to treatment initiation. If we found a matching ICTC record for a date within six months before the date of TB diagnosis, the patient was considered to have known HIV status. Although HIV status with date of testing was sometimes documented in the TB registers, we did not use the TB registers as a source of HIV status data because they were found to be incomplete at 10 of 15 TUs. We derived the following variables from the extracted data: colocation (meaning TB and HIV services available at the same facility) of either the facility at which the patient was diagnosed with or treated for TB, residence type (urban or rural), approximate distance (to the nearest kilometer) between patients’ home and the ICTC linked to the health facility providing TB treatment services, type of health facility (medical college hospital or sub-division hospital or Primary Health Center) where each patient received TB diagnosis and time gap between TB diagnosis and HIV testing (for those who got tested for HIV). The approximate distance between patient’s home and the ICTC was ascertained by the field health care workers of the general health system. If a patient utilized different facilities for diagnosis and treatment, distance between the patient’s home and the ICTC linked to the treatment facility was calculated because in all cases the treatment facility closer to the patient’s home.

We reviewed South 24 Parganas District training log registers and noted whether providers of TB services and of HIV services had participated in the training on TB/HIV integration. We reviewed the minutes of district level monthly meetings to determine what information hospital healthcare workers had been exposed to regarding TB/HIV collaborative activities. At each ICTC within South 24 Parganas, the number of HIV testing kits supplied and consumed per month was extracted from stock registers. We reviewed ICTC registers to determine the total number of patients tested for HIV each month, by patient group (e.g., referred from antenatal clinic, referred because of diagnosis of TB, referred from outpatient or in-patient wards, walk-in).

#### **Patient interview- quantitative component**

We selected a subset of patients enrolled for anti-TB treatment by simple random sampling. The sample size needed was calculated to detect an odds ratio of 1.5 (between failure to test for HIV and tested for HIV) with 80% power and 95% confidence for the variable “distance between home and ICTC more than 10 kms”. The estimated sample size of 168 was increased to 204 anticipating that 20% of patients selected would not complete the interviews either due to absence, hospitalization or refusal. We used a pre-tested semi-structured interview schedule (SSIS) and conducted interviews within a week after completion of the IP of treatment. The variables assessed were marital status, income group, literacy, occupation, referred for HIV test or not, counseled or not, category of provider who referred for HIV testing, and timing of referral.

Questionnaires for patients were translated and back-translated to the local language (Bengali) and pre-tested before use. Patient interviews were conducted by five trained interviewers in Bengali. Two supervisors cross checked the interviews for completeness and accuracy.

#### **Provider interviews and Focus Group Discussions (FGD)- qualitative component**

We held provider interviews and FGDs after we had analyzed the quantitative data available from records and patient interviews. We selected a convenience sample of Medical Officers (MO) and health workers who provided directly observed therapy to patients with TB (DOT providers) for interviews from the existing 102 health care facilities in the study district, based on 1) magnitude of referral for HIV testing and 2) presence or absence of ICTC in the health facility. At least one provider (MO or DOT provider) was chosen from each of the 15 TB units. We conducted two FGDs, one with all of the TB treatment supervisors from 15 TB units and one with all of the counselors from 14 ICTCs. Using an open ended questionnaire we asked providers about their awareness of national policy on TB/HIV program collaboration and whether they had been trained in TB/HIV program collaboration. We asked questions to assess their perceptions about the policy of routine referral of patients with TB for HIV testing and reasons for not referring patients with TB for HIV counseling and testing. We ascertained provider knowledge of recording and reporting procedures of PITC using a set of open ended questions during FGDs. The Principal Investigator (PI) conducted and recorded interviews with providers in English and conducted and recorded results of FGDs in Bengali. All Bengali transcripts of FGDs were translated to English before analysis.

### Data Handling and Analysis

Quantitative data from the data collection sheet and patient interviews were double-entered into Epi-Info software version 3.5.3 (Center of Disease Control and Prevention, Atlanta, USA), by independent data entry operators. Databases were compared and inconsistencies resolved through referral to the original data collection sheet. Statistical analysis was performed using SPSS software version 13.0. Relative risks with 95% confidence intervals (CI) were calculated for association between each exposure variable and the outcome of interest (failure to be tested for HIV within IP). Chi square (*x^2^*) tests were used for comparing proportions and p values <0.05 were considered statistically significant.

We used inductive analysis for qualitative data obtained from provider responses and FGDs (i.e., categories of analysis were not imposed *a priori* on the data but were identified through the analysis process) [9]. Provider perceptions about why patients with TB were not being tested for HIV during the IP were noted on spreadsheets. Similar answers were coded manually by the principal researcher. These codes were then grouped into emerging themes [10].

## Results

### Patient Record Review

Of 1,651 patients registered for TB treatment, 18 (1%) had documentation of HIV test performed within six months prior to the time of TB diagnosis (i.e. had known HIV status) and were excluded from the study. Of 1,633 patients with TB included in the study, 1,555 (96%) were adults (≥15 years), 1167 (72%) were males, 1,375 (84%) lived in rural areas, and 1,356 (82%) did not have history of previous anti-TB treatment. A total of 1195 (73%) patients were diagnosed with TB in primary health centers, 325 (20%) in sub-divisional hospitals and 113 (7%) in medical college hospitals (Table 1). In addition to the health facilities in South 24 Parganas District, patients were also diagnosed with TB at twenty five other health facilities in the neighboring districts. Of 1,633 patients with unknown HIV status at the time of TB diagnosis, 425 (26%) were tested for HIV by the end of IP. Of these, 362/425 (85%) were tested within the first 15 days of initiation of treatment. Of all patients tested for HIV, 8 (2%) had a positive result.

**Table 1:** Demographic and clinical characteristics of patients with TB (n = 1,633) registered for treatment from July – September, 2010 in South 24 Parganas District, West Bengal State, India.

Category	Subcategory	Number	%
**Sex**	Male	1167	71.5
	Female	466	28.5
**Age (years)**	≤15	79	4.8
	15–24	409	25.0
	25–34	312	19.1
	35–44	275	16.8
	45–54	231	14.1
	55–64	167	10.2
	≥65	160	9.8
Distance of nearest ICTC* from house (km)	<10 kms	403	24.7
	10–39 kms	1100	67.4
	>40 kms	130	8
**Residence location**	Rural	1375	84.2
	Urban	258	15.8
**History of treatment**	New	1356	83.0
	Previously treated	277	17.0
**Type of TB**	Smear Positive Pulmonary TB	1009	61.8
	Smear Negative Pulmonary TB	353	21.6
	Extra Pulmonary TB	271	16.6
**Location of TB diagnosis**	Primary Health Centers	785	48.1
	Primary Health Centers having a Tuberculosis Unit Headquarter	410	25.0
	District/Sub Divisional Hospital	59	3.7
	District/Sub Divisional Hospital with Tuberculosis Unit Headquarter	266	16.2
	Medical College Hospital	113	6.9
**HIV testing status**	Not tested during Intensive Phase (IP)	1208	74.0
	Tested during Intensive Phase	425	26.0
**Timing of HIV testing (n = 425** ** **)**	≤15 days of TB treatment initiation	362	85.2
	>15 days but within IP of TB treatment	63	14.8

ICTC = Integrated Counseling and Testing Centre (HIV testing centers),

** only for those who got HIV tested during the intensive phase.

Relative to patients diagnosed with or treated for TB at a facility with collocated ICTC, risk of failure to be tested for HIV were higher for patients diagnosed with or treated for TB in facilities without a co-located ICTC (RR 1.27, 95% CI 1.20–1.35). Age, sex, positive sputum test result and history of prior TB treatment were not associated with failure to receive testing for HIV (Table 2).

**Table 2:** Factors associated with failure to test for HIV among patients with TB in South 24 Parganas District, West Bengal State, India, July-September 2010.

Category	Failed to test for HIV during IP*(n = 1208)	Tested for HIV in IP*(n = 425)	Relative Risk	95% Confidence Interval	P Value
	n (%)	n (%)			
**Age in years**					
0–14	55 (69.6)	24 (30.4)	0.90	0.76–1.06	0.19
15–24	292 (71.4)	117 (28.6)	0.92	0.83–1.02	0.14
25–34	222 (71.2)	90 (28.8)	0.92	0.82–1.02	0.14
35–44	212 (77.1)	63 (22.9)	0.99	0.90–1.11	0.92
45–54	181 (78.4)	50 (21.6)	1.01	0.91–1.13	0.84
55–64	122 (73.1)	45 (26.9)	0.94	0.83–1.07	0.35
Above 65	124 (77.5)	36 (22.5)	**REF**		
**Sex**					
Male	872 (74.7)	295 (25.3)	1.04	0.97–1.11	0.27
Female	336 (72.1)	130 (27.9)	**REF**		
**Co-location of TB and HIV testing services**					
Not Co-located	672 (83.0)	138 (17.0)	1.27	1.20–1.35	**<0.01**
Co-located	536 (65.1)	287 (34.9)	**REF**		
**Type of TB**					
Smear Positive Pulmonary TB	736 (72.9)	273 (27.1)	0.94	0.87–1.01	0.13
Smear Negative Pulmonary TB	262 (74.2)	91 (25.8)	0.96	0.88–1.05	0.34
Extra Pulmonary TB	210 (77.6)	61 (22.4)	**REF**		
**History of previous treatment**					
New	1002 (73.9)	354 (26.1)	0.99	0.92–1.07	0.88
Previously treated	206 (74.4)	71 (25.6)	**REF**		

* IP = Intensive phase of TB treatment.

### Review of Training Register and HIV Testing Kit Stock Registers

The RNTCP district program manager, NACP district program manager, all MOTCs, all TB supervisors at the 15 TUs, and all counselors of 14 ICTCs were found to have been formally trained using the standard TB/HIV training modules. Other medical officers and healthcare workers were not trained formally but were informed of the national policy of ‘routine offer of HIV testing for all patients with TB’ in their regular monthly meetings. From July through September 2010, there were no test kits available at 6 of 14 ICTCs for periods ranging from 10 to 20 days. However, at the district level, there were more test kits distributed to ICTCs than were used.

### Patient Interviews

Of the 204 patients randomly selected, 169 (83%) were interviewed, 25 were unavailable even after three attempts, and 10 died before they could be interviewed. Of 169 patients interviewed, six were children under 15 years so we interviewed the parent who accompanied the children during the TB diagnosis process. There was no statistically significant difference between patients who were interviewed compared to all patients enrolled by age group, sex, completion of HIV testing during IP, residence type or history of previous treatment (data not shown). Of 169 patients interviewed, only 67 (40%) reported that they were referred for HIV testing and of these, 53 (79%) went to an ICTC. Of the 53 patients who went to an ICTC, 47 (89%) were tested for HIV and 6 (11%) were not. Of the 47 patients tested for HIV, 25 (47%) reported that they were counseled prior to being tested. Of 67 patients who reported that they were referred for HIV testing, 41 (61%) reported they were not informed of the purpose of the test by the TB care provider.

Of the six patients who reported that they went to ICTC after being referred for testing but did not get tested, two (33%) reported that they did not get tested because the ICTC was closed and four (66%) reported that there were no counselors to conduct HIV counseling or testing. Of the 14 persons who were referred but did not go to ICTC, eight (57%) reported they thought that the test was not necessary, four (29%) reported that the ICTC was too far and two (14%) reported that they were told by the TB program staff to go for HIV testing at another time because testing kits were not available but did not go for testing at a later date. Of these 14 persons referred to ICTC but who did not go, 3 were referred from a facility that was co-located with an ICTC. In total, 122/167 (72%) of interviewed patients were not tested for HIV during the IP of anti-TB treatment (Table 3).

**Table 3:** Patient characteristics and HIV test referral outcomes as reported in interviews with patients with TB (n = 169) registered for treatment between July to September 2010, South 24 Parganas District, West Bengal State, India.

Patient Characteristics	Number	%
**Marital Status**		
Married	136	80
Unmarried	33	20
**Income group** (INR per month)*		
<4000	144	85
>4000	25	15
**Literate**	104	62
**Occupation**		
Working	92	54
Jobless	31	18
Student	12	7
Housework	34	20
**Referral for HIV testing**		
Not referred	102	60
Referred	67	40
**Reaching HIV testing centre (N = 67** # **)**		
Did not go to ICTC** after referral	14	21
Went to ICTC after referral	53	79
**HIV testing (N = 53** ## **)**		
Not tested	6	11
Tested	47	89

* 100 INR (Indian rupees) = 2.2 USD (US Dollars) as on 1^st^ September 2011,

** ICTC = Integrated Counseling and Testing Centre (HIV testing centers);

# only among those who were referred;

## only among those who reached ICTC.

Among patients who reported that they were referred for HIV testing, patients who reported being referred by a DOT worker had higher risk of not undergoing HIV testing relative to patients who reported being referred by a Medical Officer (RR 3.53, 95% CI 1.62–9.57). We found that among patients referred by DOT workers 12/29 (41%) were exposed to co-located facilities compared to 22/38 (58%) of patients referred by Medical. Patients referred for HIV testing during anti-TB treatment were less likely to get tested relative to patients referred at the time of diagnosis (RR 2.91, 95% CI 1.19–7.10), (Table 4)**.** Patients’ marital status, literacy, occupation and income were not significantly associated with failure to get HIV tested. Among patients diagnosed with TB, 43% (34/79) of patients diagnosed in a facility with a co-located ICTC were referred for HIV testing compared to 36% (33/90) of patients diagnosed at a facility without a co-located ICTC.

**Table 4:** Factors associated with failure to be tested for HIV among patients with TB who reported being referred for HIV testing after TB diagnosis was made in South 24 Parganas District, West Bengal State, India – July–September, 2010.

Category	Failed to be tested for HIV in IPn = 20	Tested for HIV during IP*n = 47	Relative Risk	95% Confidence Interval	P Value
	n (%)	n (%)	–	–	–
**Referring Person**					
Referral by DOT workers	15 (52)	14 (48)	3.53	1.62–9.57	**<0.01**
Referral by Medical Officers	5 (13)	33 (87)	REF		
**Time of Referral**					
During TB treatment	15 (44)	19 (56)	2.91	1.19–7.10	**<0.01**
At TB diagnosis	5 (15)	28 (85)	REF		
**Method of Referral**					
Referral form not used	15 (36)	27 (64)	1.79	0.74–4.32	0.17
Referral form used	5 (20)	20 (80)	REF		

* IP = Intensive phase of TB treatment.

### Provider Interviews and Focus Group Discussions (FGD)

Key informant interviews were conducted with 16 MOs and four DOT providers. More than half (13/20) of providers reported awareness of the policy to refer all patients with TB for HIV testing and 35% (7/20) reported that program managers of both the national TB control and AIDS control programs provided minimal supervisory support. Almost all providers (17/20) believed that patients would not undergo HIV testing even when recommended by a provider. Most providers (15/20) reported that they had not been formally trained using standard-HIV training modules, and that they do not regularly refer all patients with TB for HIV testing. We learned that providers do not refer for HIV testing because they believe that ICTCs are not co-located with TB facilities, that they are too far for patients to travel especially in view of poor public transport facilities, and that ICTCs provided irregular service and frequently suffered from HIV testing kit shortages (Table 5). Providers felt that very few patients were likely to be HIV positive and therefore questioned the rationale for referring patients for testing.

**Table 5:** Perception of Providers on barriers to HIV testing for people with TB in South 24 Parganas District, West Bengal, India.

Barriers	Providers Citations	Type of Respondent
**Lack of knowledge on TB-HIV guidelines**	***“*** *Those TB patients who have high risk behavior need to be tested for HIV”*	(Medical Officer of a sub-divisional Hospital with ICTC*)
	*“Those who are not responding to TB treatment need to be tested for HIV”*	(Medical Officer of a Health centre without ICTC)
	*“Is there any Government order to send TB patients for HIV testing?”*	(Medical Officer of a Health centre without ICTC)
**Perception that ICTC is too far for patients to go**	*“Patients do not want to go to ICTC leaving their work”*	(Medical Officer of a Health centre with ICTC)
	*“ICTC is very far from here. This is a river delta island”*	(Medical Officer of a Health centre with ICTC)
**Perception that ICTC is not** **providing patient friendly services**	*“Patients are made to wait a long time. Sometimes they complain of rude behavior. They are made to come again and again”*	(DOT Provider of a sub-divisional Hospital with ICTC)
	*“ICTC is not open regularly. They are monitored from higher level and* *we do not know much about their functioning”*	(Medical Officer of a sub-divisional Hospital with ICTC)
	*“ICTC is too crowded as ante natal mothers are also tested here.* *Patients get their results after a week”*	(TB treatment supervisor of one TB Unit)
	*“No one knows what the ICTCs do. No one monitors their work and* *they do not listen to anyone”*	(Medical Officer of a sub-divisional Hospital with ICTC)
**Perception that there is shortage** **of testing kits**	*“Kits are short supply I heard, we are told not to refer too many patients”*	(Medical Officer of a Health centre with ICTC)
**Perception that patients will not undergo testing if informed the** **test is for HIV**	*“Patients do not understand what is HIV”*	(Medical Officer of a Health centre with ICTC)
	*“I don’t think they will go for the testing if they know it is for HIV”*	(Medical Officer of a Health centre with ICTC)
	*“They may not get the test done once they know they have already* *been diagnosed of TB”*	(Medical Officer of a Health centre with ICTC)

* ICTC = Integrated Counseling and Testing Centre (HIV testing centers).

In FGD, TB treatment supervisors indicated that they had received training on TB/HIV guidelines but were not yet conversant with correct reporting procedures. They also shared concerns about the shortage of HIV testing kits and felt that physicians were apathetic towards referral of HIV testing for patients with TB. ICTC counselors indicated that they receive few referrals for HIV testing from TB treatment providers, minimal supportive supervision from both TB and HIV programs, and intermittent stock outs of kits.

## Discussion

Nearly three-fourths of patients with TB in South 24-Parganas district, West Bengal, were not tested for HIV during the intensive phase of TB treatment. Based on our data we believe that a key reason that patients were not tested for HIV was that they were not routinely referred for testing by TB treatment providers. We found that testing for HIV was strongly associated with receiving TB treatment or diagnostic services at a facility that had on-site HIV testing services; however at South 24-Parganas facilities both with and without on-site HIV testing services patients reported low rates of referral for HIV testing. Providers indicated that they often do not refer patients for HIV testing because they believe that patients will not present for HIV testing because ICTCs are located too far away, or because HIV testing kits are not consistently available at ICTCs. We also found that health system factors rather than patient factors are responsible for low levels of testing. Several HIV testing centers had intermittent stock-outs of test kits during the study period, though the total number of HIV test kits at district level was adequate compared to the number used at ICTC, suggesting that supply chain management was sub-optimal. Though national policy is to provide training on TB/HIV to all health care providers, formal training was provided only to a few key staff of RNTCP and NACP. In contrast to studies in South India [11], East Asia [12]–[14] and African countries [15]–[18], our study in an Eastern India district showed that failure to test was not associated with any demographic or clinical characteristics of patients.

Our findings support the importance of co-location of HIV testing and TB care services as demonstrated in some Asian [12]–[14] and African [19], [20] countries. Through our study we were also able to demonstrate the importance of HIV test kit supply chain management. Most importantly, our study highlights provider knowledge, beliefs, and practices that may be a barrier to HIV testing for patients with TB. Similar provider beliefs have been reported in Uganda [15], Tanzania [21] and Ukraine [22].

In Karnataka, a state in South India, the uptake of HIV testing was 84% in the same period as this study [3]. However, Karnataka is a state with high HIV prevalence and with decentralized and widely available HIV testing services. In contrast, West Bengal has much lower HIV prevalence and fewer HIV testing services. HIV awareness among the community in West Bengal remains lower than reported in states of India that have higher HIV prevalence [23]. Our findings that at least half of patients with TB were not referred for HIV testing, were not informed what the test was for, and were not counseled prior to HIV testing indicates there are gaps between national policy and local implementation. One explanation for the observed low levels of provider knowledge and poor referral practices is that West Bengal has a relatively low HIV prevalence so providers there may have less experience in caring for patients with HIV infection just as in other low prevalence settings [24], [25].

Increasing ease of access to HIV testing can increase uptake of PITC. Examples of strategies to increase HIV testing among patients with TB that have been successfully implemented in other settings include provision of both TB and HIV services by a single health provider as in Kwazulu-Natal and co-locating TB treatment and HIV testing services as in Khayelistha, South Africa [26]. Robust logistic supply-utilization management may be used to prevent even brief shortages of HIV testing kits as demonstrated in Uganda [15]. Increasing community awareness of the link between TB and HIV may also increase testing for HIV. In Kenya, an increase in HIV testing from 32% to 59% was observed when patients with TB were provided health education before the national scale-up of routine HIV testing. Based on our data and other research, we believe that TB/HIV training for all appropriate staff, supportive supervision, and effective supply management of HIV test kits can improve HIV testing among people with TB [27].

Our study had several limitations. Information on referral of patients for HIV testing by TB care providers was based on patients’ self-report. Although we cannot exclude possible recall or reporting bias, particularly according to whether or not a patient was referred for testing, our finding of low rates of referral for HIV testing is consistent with information from our provider interviews and FGDs. Although facility meeting minutes provided evidence of sensitization of providers to HIV testing of patients with TB, were not able to assess the proportion of providers sensitized. We also could not verify all subjective claims made by providers, for example that ICTCs have staff shortages and irregular hours of operation. Addressing this limitation in future studies would provide an evidence base to recommend mitigation of some of these issues ascertained. Though the present study was conducted in one district, challenges identified in this study are likely to be common to other districts of West Bengal state, which are also in the initial phase of implementing HIV testing among patients with TB. Thus these study findings will help in formulating and prioritizing operational procedures to strengthen existing TB/HIV collaborative activities in the state. Because TB/HIV collaborative activities are being implemented in a phased manner in India, results from this study may inform activities as they are implemented and scaled up in other parts of India.

Universal HIV testing in persons with TB has not yet been achieved in West Bengal. This represents a missed opportunity for HIV testing, counseling and ART services which in turn prevent excess morbidity and mortality associated with HIV infection in patients with TB [28]–[30]. Comprehensive strategies to change providers’ beliefs regarding referral for HIV testing of all people with TB, formal training of all providers in TB/HIV collaborative activities, decentralization of HIV testing to all TB care centers or direct provision of HIV testing by TB clinic staff, and improved HIV test kit supply chain management may increase uptake of HIV testing among patients with TB.
